# International exchange and collaboration in science

**DOI:** 10.1093/nsr/nwz150

**Published:** 2019-10-12

**Authors:** Mu-ming Poo

**Affiliations:** 1 CAS Center for Excellence in Brain Science and Intelligence Technology, China; 2 Executive Editor-in-Chief of NSR

Scientific exchange and collaboration between the two largest economies in the world are now facing serious challenges, due to isolationist policies of the Trump administration. The USA has been the leading country in attracting international talents and in promoting scientific exchange and collaboration. The sudden change in the climate appears to be triggered by the USA–China trade conflict and the perceived threat of China’s scientific and technological advancement to the US supremacy in the world. Under the disguise of national security, the Trump administration has ordered the Federal Bureau of Investigation to step up the surveillance and investigation on research activities of foreign-born students, researchers and visiting scholars in US research institutions, targeting specifically those of Chinese origin, on the basis of a few isolated cases of alleged illegal transfer of intellectual property.

In response to the ill-conceived practices of law enforcement and intelligence agencies, 59 US scientific organizations, representing hundreds of thousands of scientists, engineers and educators around the world, issued a joint position statement on 4 September to the leaders of four major government funding agencies, raising ‘the escalating concern that new policies and procedures under consideration to minimize security risks will have unintended effect of harming the scientific enterprise’. The statement mainly alerts the government about long-term consequences of these practices on US research institutions in attracting and retaining foreign talents, but an immediate consequence will be the impediment of scientific exchange and collaboration between the USA and China. Fewer Chinese scientists are now attending conferences or visiting research institutions in the USA, and fewer collaborations with US scientists are being initiated. Fruitful collaborations between Chinese and US scientists in the past, as exemplified by the Daya Bay Neutrino Experiment, are likely to be severely affected.

The dark cloud over scientific exchange and collaboration between the USA and China, however, had little effect on the flourishing activity between China and many other countries. At our CAS Center for Excellence in Brain Science and Intelligence Technology alone, nine collaborative projects involving scientists from six different countries have been initiated in the past two years, and such activity is on the rise in most research institutions across China. In mid-September, organizers of major brain projects around the world, including representatives from the US BRAIN Initiative and private foundations, gathered in Daegu, Korea for a special forum of the International Brain Initiative (IBI) during the 10th Congress of the International Brain Research Organization. The main goal was to organize international platforms for data sharing, through which brain researchers around the world could collaborate more effectively in exploring the frontier of neuroscience and in developing treatments for major brain diseases that are inflicting all societies. The IBI discussion focused on how to develop mechanisms that could ensure equitable credit attribution and data sharing that would benefit all participants, reflecting the desire of neuroscientists around the world for scientific exchange and collaboration.

Even under the pressure from the federal government, researchers at the US universities remain enthusiastic in USA–China scientific exchange and collaboration, particularly in critical fields such as climate change. On 23 September, the former California Governor Jerry Brown and Zhenhua Xie, China’s top climate official, announced in Sacramento, California the establishment of the California-China Climate Institute, to be housed at the University of California, Berkeley in partnership with Tsinghua University in Beijing. The Institute will work on zero-emission vehicles and low-carbon transportation, carbon pricing, climate adaptation, sustainable land use and agriculture, carbon capture and storage, and long-term climate policy enforcement. It will also train researchers from both universities and host a series of ‘subnational climate dialogues’ among business, government and climate research leaders in the USA and China.

Active fields of scientific inquiry are often accompanied by intense competition among scientists, who consider getting there first with the signature of personal achievement. Competition could accelerate the progress of science by focusing the scientists’ energy and propelling their creativity. But competition should not be a hindrance to exchange and collaboration, which can benefit both sides of the competition. Indeed, competitors often communicate extensively in conferences and sometimes end up collaborating, when they foresee that the common goal could not be achieved by working alone. It is all too apparent that many serious problems facing the world, from global warming to infectious diseases, can only be solved through exchange and collaboration among scientists around the globe. An isolationist view of the world, in economy or in science, runs against the spirit of the global village, in which all countries are bound by a common fate. The dark cloud covers only a small part of the bright sky.

